# Retraction: Effects of the COVID-19 Pandemic on Stroke Patients

**DOI:** 10.7759/cureus.r22

**Published:** 2021-01-13

**Authors:** Hammad Ghanchi, Ariel Takayanagi, Paras Savla, Omid R Hariri, Emilio C Tayag, Michael Schiraldi, Lucille Jorgensen, Dan E Miulli

**Affiliations:** 1 Neurosurgery, Riverside University Health System Medical Center, Moreno Valley, USA; 2 Department of Neurosurgery, Kaiser Permanente-Orange County, ANAHEIM, USA; 3 Neurology and Neurosurgery, Desert Regional Medical Center, Palm Springs, USA; 4 Neurosurgery, Desert Regional Medical Center, Palm Springs, USA; 5 Neurosurgery, Redlands Community Hospital, Redlands, USA; 6 Stroke Program Coordinator, Arrowhead Regional Medical Center, Colton , USA; 7 Neurosurgery, Arrowhead Regional Medical Center, Colton, USA

This article has been retracted as it includes data published without permission from the Get With the Guidelines database, which is property of American Heart Association (AHA). Although the authors' hospital had access to this database and they did receive IRB approval, the terms of use between the hospital and AHA do not allow publication of the data in a public sphere. The authors were hoping to receive retrospective approval from the AHA but have not been successful in getting this permission thus far. As a result this article has been retracted at the request of the AHA and with full agreement by the authors.

